# Nuclear Receptor Regulation of Aquaglyceroporins in Metabolic Organs

**DOI:** 10.3390/ijms19061777

**Published:** 2018-06-15

**Authors:** Matteo Tardelli, Thierry Claudel, Francesca Virginia Bruschi, Michael Trauner

**Affiliations:** Hans Popper Laboratory of Molecular Hepatology, Division of Gastroenterology & Hepatology, Internal Medicine III, Medical University of Vienna, Währinger Gürtel 18-20, A-1090 Vienna, Austria; matteo.tardelli@meduniwien.ac.at (M.T.); thierry.claudel@meduniwien.ac.at (T.C.); francesca.bruschi@meduniwien.ac.at (F.V.B.)

**Keywords:** aquaporins, nuclear receptors, glycerol, metabolism

## Abstract

Nuclear receptors, such as the farnesoid X receptor (FXR) and the peroxisome proliferator-activated receptors gamma and alpha (PPAR-γ, -α), are major metabolic regulators in adipose tissue and the liver, where they govern lipid, glucose, and bile acid homeostasis, as well as inflammatory cascades. Glycerol and free fatty acids are the end products of lipid droplet catabolism driven by PPARs. Aquaporins (AQPs), a family of 13 small transmembrane proteins, facilitate the shuttling of water, urea, and/or glycerol. The peculiar role of AQPs in glycerol transport makes them pivotal targets in lipid metabolism, especially considering their tissue-specific regulation by the nuclear receptors PPARγ and PPARα. Here, we review the role of nuclear receptors in the regulation of glycerol shuttling in liver and adipose tissue through the function and expression of AQPs.

## 1. Introduction

Glycerol is a necessary constituent of triglyceride (TG) backbones. TGs are the main source of energy storage for the human body, taking part in metabolic processes such as fatty acid oxidation, and the biosynthesis of other lipid molecules and lipoproteins [1]. The regulation of their metabolism is finely tuned by nuclear receptors (NRs), a family of transcriptional regulators involved in diverse functions such as glucose metabolism, lipid homeostasis, and development [2]. NRs play key roles in cell differentiation and metabolism, being connected with numerous pathologies such as cancer, liver steatosis, inflammation, fibrosis, and cholestasis. The huge network of target proteins associated with the action of NRs is tremendously complex, including many co-regulators and other micro and long noncoding RNAs. Aquaporins are a family of membrane water channels, which are involved in a wide range of physiological functions and diseases. They mediate osmotic water transport across cell plasma membranes and glycerol diffusion, regulating pivotal physiologic functions such as cell proliferation and lipid metabolism. Since glycerol shuttling mediated by aquaporins and NR transcriptional activities are strong players in regards to TG and fatty acid metabolism, we review their interplay in this article.

## 2. Nuclear Receptor Functions and the Regulation of AQPs

The NR superfamily includes 48 transcription factors in humans (49 in mice) [3]; in this review, we mainly focus on the peroxisome proliferator-activated receptors (PPARs), the farnesoid X receptor (FXR), and the liver X receptors (LXRs). The PPAR family comprises three members: PPARα (*NR1C1*), PPARδ (*NR1C2*), and PPARγ (*NR1C3*). They usually form a heterodimer with the retinoid X receptors (RXRs) to regulate gene expression. PPARα is highly expressed in the liver, heart, kidney, and skeletal muscle, and senses fatty acids. Its functional role in the liver is to maintain lipid homeostasis, promoting fatty acid oxidation and also triggering the formation of ketone bodies [4]. In addition to its metabolic actions, PPARα plays also an anti-inflammatory role in immune cells, and can be activated by the hypotriglyceridemic drugs belonging to the fibrates class [5]. PPARδ is ubiquitously expressed, and promotes fat burning and weight loss in mice. Treatment of obese mice with a PPARδ agonist improved insulin sensitivity, while PPARδ knockout mice showed reduced energy expenditure, perhaps because PPARδ regulates mitochondrial oxidative metabolism and thermogenesis through peroxisome proliferator-activated receptor-gamma coactivator 1-alpha (PGC-1α) [6]. PPARγ is well known as the master regulator of adipogenesis, and is mostly expressed in adipose tissue, and to a smaller extent in skeletal muscle and liver tissue [7], and can be activated pharmacologically by the anti-diabetic drugs, thiazolidinediones, which improve insulin sensitivity by promoting fatty acid (FA) storage in adipocytes [8]. FXR (*NR1H4*) has four different isoforms, FXRα1, FXRα2, FXRα3, and FXRα4, expressed at highest levels in the intestine and liver. In addition, rodents and dogs express FXRβ (*NR1H5*) mainly in testis, a nuclear receptor closely related to FXRα, and probably activated by lanosterol [9]. However, FXRβ remains a pseudogene in humans and primates, and hence, does not play a role in aquaporin regulation [9]. FXR, like PPARs, forms heterodimers with RXR to trigger gene expression [10,11]. Importantly, FXR regulates bile acid synthesis by repressing the expression of the cholesterol 7α-hydroxylase gene (*CYP7A1*), the rate-limiting enzyme in the biosynthesis of bile acids from cholesterol [12,13]. FXR, which is a bile-acid sensor, directly reduces not only serum high-density lipoprotein (HDL)-cholesterol and triglyceride levels, but also increases hepatic glycogen synthesis. Moreover, FXR indirectly induces liver protein synthesis, and controls metabolic rate and insulin sensitivity, by inducing *FGF19* expression in humans, or *Fgf15* in rodents [14]. The LXR family also has two different isoforms, LXRα (*NR1H3*) and LXRβ (*NR1H2*), which are of importance for the regulation of cholesterol levels in the body. These nuclear receptors are endogenously activated by modified forms of cholesterol known as oxysterols, and control the expression of genes important for lipid metabolism and cholesterol uptake, efflux, transport, and excretion in multiple tissues [15]. PPARγ, together with LXR, was also shown to be involved in a signaling pathway controlling differentiation of monocytes, modulating macrophage activation through several anti-inflammatory mechanisms [16].

NRs are known to regulate pivotal components of human metabolism, such as the lipid metabolism machinery in liver and adipose tissue, as well as glycerol shuttling, mediated by aquaglyceroporins.

Aquaporins (AQPs) are a family of 13 known different channel proteins, which facilitate the passive transport of water across the plasma membrane in response to osmotic gradients created by the active transport of solutes. AQPs can be categorized based on their solute transport selectivity into two main groups; orthodox AQPs facilitate the passage of water and small solutes, whereas aquaglyceroporins also facilitate the passage of glycerol [17,18]. Aquaglyceroporins, namely AQP3, AQP7, AQP9, and AQP10, were shown to transport water, glycerol, and other small polar solutes, gases, amino acids, sugars, and arsenite [19]. AQPs are expressed in various organs and tissues of special interest, such as the liver, immune cells, or adipocytes, with several functions depending on their localization [19,20] (summarized in Table 1). AQPs share 25% to 60% protein sequence homology, and are structurally organized in homo-tetrameric complexes, where each monomer contains six transmembrane segments, together forming a pore. Most AQPs are localized across the lipid bilayer of the cell plasma membrane, and their expression is mainly regulated at transcriptional level. They were shown to translocate to the plasma membrane upon certain stimuli from intracellular vesicles, thus modulating its permeability and cell functions [21]. The relationship among AQPs, glycerol transport, and lipid metabolism was investigated in mice, and identified as important in many physiologic processes and disease models [22,23] (Table 1).

## 3. Aquaporin Regulation in Adipose Tissue

Adipose tissue (AT) is one of the largest glycerol reservoir in the human body. Many studies demonstrated the expression of aquaporin 7 (AQP7) in both white and brown AT, with the highest expression in visceral white adipose tissue (vWAT) [44]. AQP7 was shown to be key in adipocyte metabolism, as well as playing a pivotal role in insulin resistance and lipid/glucose homeostasis. AQP7-deficient mice had severe hypoglycemia characterized by low plasma glycerol levels and impaired glycerol release during fasting, highlighting the pivotal role of glycerol shuttling from adipocytes to the liver during gluconeogenesis [45]. Other authors demonstrated that the impaired glycerol efflux in AQP7 knockout (KO) mice triggered accumulation of TGs in adipocytes (since glycerol is needed as a backbone for TG biosynthesis), resulting in adipocyte hypertrophy and weight gain [35]. Insulin resistance was also observed in AQP7 KO mice as they aged, with increased fasting plasma glucose and insulin concentrations. In addition, AQP7 KO mice, fed on a high fat/high sucrose diet, demonstrated early onset of obesity and insulin resistance even at a young age [36]. AQP7 was shown to be a direct target of PPARγ, as evidenced in in vitro studies in 3T3-L1 cells, and in mice treated with pioglitazone [46]. Moreover, in another elegant molecular study, the authors demonstrated the direct binding of the PPARγ and RXRα complex to the AQP7 peroxisome proliferator response element (PPRE) [47]. Shortly after, another group established that PPARα deficiency blocked the increase in the AQP7 transcription level in AT, triggered by fasting (typically mediated by PPARα) [48]. An interesting hormonal regulation of AQP7 by estrogens in adipocytes was also shown in a recent study. The authors measured the amount of AQP7 in ovariectomized mice, discovering a tight reduction in AQP7 levels in visceral fat, which could be restored with estradiol supplementation [49].

AQP3 was also found to be expressed in AT, namely in the stroma vascular fraction of omental, subcutaneous AT, and also in freshly isolated adipocytes [50]. Interestingly, insulin stimulation profoundly increased AQP3, AQP7, and AQP9 expression in human adipocytes, whereas leptin had opposite effects via the phosphatidylinositide 3-kinase (PI3K) signaling cascade [50]. Leptin administration in obese (ob/ob) mice strongly downregulated AQP3 and AQP7 in AT, while upregulating hepatic AQP9, perhaps to coordinately prevent lipid accumulation in AT and the liver during obesity [51]. In a recent study in which obese Wistar rats were subjected to sleeve gastrectomy, AQP7 levels were found upregulated in epididymal and subcutaneous fat, together with AQP3, whereas hepatic AQP9 remained unchanged, contributing to reduced liver steatosis and weight loss [52]. AQP9 was also shown to work together with AQP3 during lipolysis to facilitate glycerol efflux [53]. Genetic studies revealed that a PPARγ polymorphism, namely a cytosine (C) to thymine (T) substitution in exon 6, nearly doubled circulating leptin levels in obese mice [54]. It would be of interest to measure AQP3, AQP7, and AQP9 expression in these patients, and determine whether PPARγ sequence variations could contribute to modifications in glycerol disposal, specifically in obese mice, and how glitazone treatment would affect it. In addition, since PPARγ agonists are repressors of leptin expression, molecular studies to understand how direct PPARγ regulation versus leptin-mediated regulation of aquaporins can take place in vivo would also be interesting to decipher the time course, and hence, the stimuli controlling aquaporin activities [55].

Moreover, another aquaporin specifically expressed in adipocytes was recently discovered—AQP10. AQP10 might also take part in glycerol metabolism, and is potentially connected to metabolic dysfunctions such as obesity [56], leading to the hypothesis that a co-functional redundancy of aquaporin channels facilitating the diffusion of glycerol in AT could exist [53]. Since aquaporin 10 is a newcomer in the field, molecular studies of its regulation by PPARs and adipokines, such as leptin, are eagerly awaited to further understand the relative contributions of the various aquaporins, and whether it is only an alternative transporter [56]. In a recent report, authors speculated on the localization and function of AQP11 in AT, discovering the presence of AQP11 in both subcutaneous and visceral human mature adipocytes, with a preferential localization in proximity of lipid droplets [57].

## 4. Aquaporin Regulation in Liver

**Figure 1:**
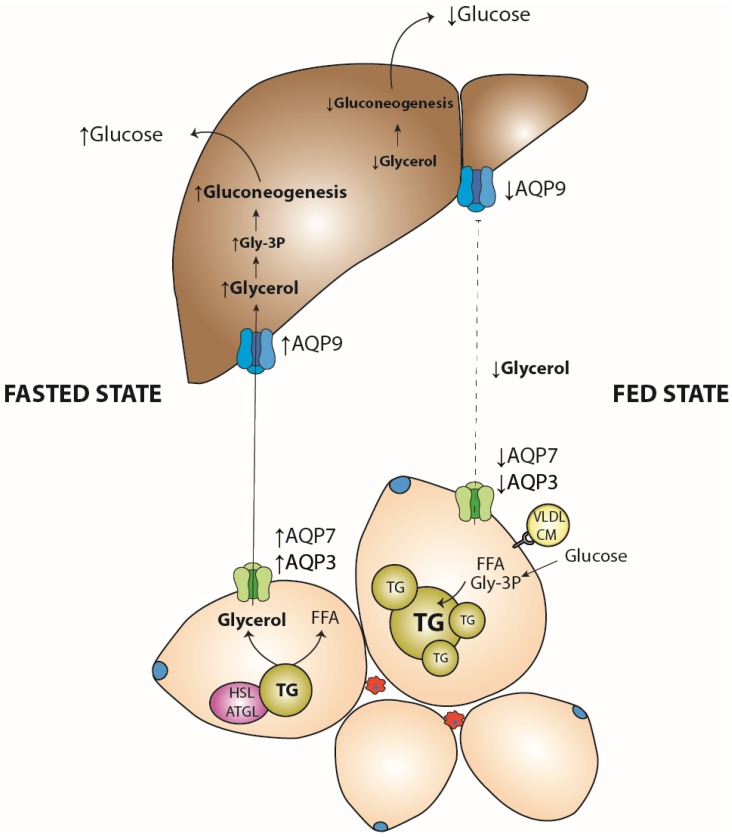
**Regulation of adipose tissue aquaporins (AQPs), AQP3/AQP7, and liver AQP9 during fasting and feeding states.** Fasting induces lipolysis in adipocytes, and gluconeogenesis in liver (as depicted on the left side of the figure); AQP7 and AQP3 messenger RNA (mRNA) levels are elevated in adipose tissue (AT), facilitating glycerol release into the blood stream. In turn, AQP9 mRNA levels in liver are also increased in order to transport glycerol in the portal vein. In the liver, glycerol is transformed into glycerol 3-phosphate (Gly-3P), and used as a substrate for gluconeogenesis, increasing glucose levels in the blood. In the fed state, the abundance of nutrients triggers a metabolic switch from lipolysis to lipogenesis in adipocytes, suppressing glucose production in the liver. Thus, adipose AQP3 and AQP7 mRNA levels decrease, and free fatty acids (FFA) are stored in the form of triglycerides (TGs). Circulating glycerol levels also decrease in parallel with the reduction in adipose AQP-mediated glycerol shuttling, and liver AQP9 levels. HSL—hormone-sensitive lipase; ATGL—adipose triglyceride lipase; VLDL—very low-density lipoprotein; and CM—chylomicrons. Increased (↑) decreased (↓).

AQP1, AQP3, AQP7, AQP8, and AQP9 are expressed in human liver with different cellular distributions [58]. More specifically, AQP3 expression is mainly localized in Kupffer cells, AQP7 in hepatocytes and liver endothelial cells, and AQP9 in cholangiocytes. Moreover, AQP1 was also found in endothelial cells, and AQP8 and AQP9 were found in hepatocytes [58,59]. AQP9 is expressed in multiple organs, but especially abundant in the liver, playing a substantial role in hepatic glycerol and glucose metabolism [60]. During fasting, AQP9 favors glycerol entrance into the hepatocytes in mice [61]. Therefore, glycerol released through AQP7 in the adipose tissue is taken up by the liver via AQP9 for glucose neo-synthesis [62]. Under feeding conditions, a rise in plasma insulin concentration results in the suppression of lipolysis and the reduction in adipose AQP7 and liver AQP9 expression, thus decreasing glycerol release from adipocytes and liver gluconeogenesis [63]. The coordination of these two aquaporins, located in adipose tissue and the liver, respectively, seems to be pivotal in glucose metabolism under physiological conditions, as well as in obesity and type 2 diabetes mellitus (T2DM) [62] (mechanism summarized in Figure 1). Such coordinated glycerol release and uptake, controlled by PPARα in the liver and PPARγ in adipose tissue, reinforces that PPARs are sensors involved in global lipid homeostasis. Interestingly, the vWAT expands in obese and diabetic patients. Gene profiling showed that, in contrast to subcutaneous WAT, vWAT expressed more insulin receptors lacking exon 11, which is known to decrease insulin sensitivity, thus making this fat depot prone to insulin resistance. Moreover, leptin expression is significantly lower in vWAT, whereas PPARγ expression was reduced only in patients with a body mass index (BMI) lower than 30 [64]. It would be, therefore, important to measure how glycerol turnover and aquaporin expression are affected by both leptin and PPARγ expression in vWAT of obese and type-2 diabetics, in order to limit abnormal gluconeogenesis, either by impacting their vWAT, PPARγ, or hepatic PPARα signaling. Several PPARγ isoforms (gamma 1, 2, and 3) exist, and have different functions [65]. Whereas PPARγ1 is mainly expressed in inflammatory cells, and PPARγ2 in WAT during fatty liver development, PPARγ2 expression is induced in hepatocytes [66]. It is, therefore, also of interest to determine whether the induction of PPARγ2 expression at different stages of fatty liver development would help to pharmacologically modulate aquaporin expression, and restore glycerol homeostasis, especially knowing that polymorphisms, such as the proline to alanine at position 12 (Pro12Ala), are important modulators of the PPARγ2 response, depending on the metabolic context [67].

The role of AQPs in water transport and bile formation is also important. Bile serves as an emulsifier of dietary lipids, and its secretion is a complex process of concerted canalicular water secretion in response to osmotic gradients originated from the active transport of solutes [68]. Bile flow is, therefore, strictly dependent on water secretion in the canalicular lumen, driven by AQPs. In fact, a role of AQP8 in biliary water transport was shown in hepatocytes. In this study, Larocca et al. silenced the *AQP8* gene in HepG2 cell lines via RNA interference, discovering a significant reduction in canalicular volume in *AQP8*-silenced cells, followed by inhibition of water transport in response to several stimuli, such as an inward osmotic gradient, and a bile secretory agonist. These data suggest that AQP8 is a rate-limiting channel aimed at water secretion in canalicular membranes during bile secretion [69]. Moreover, AQP8 canalicular expression and translocation were strongly induced by glucagon via an increase in hepatocyte-membrane water permeability due to protein kinase A [70]; whereas AQP9 was shown to be involved in the sinusoidal uptake of water [71]. AQPs were also demonstrated to play diverse roles in a cell-specific dependent manner. Hepatocytes express AQP0, AQP8, AQP9, and AQP11 [72,73], mediating water transport and glycerol shuttling. Expression of AQP9 in hepatocytes was shown to be regulated by PPARα, which preferentially directed glycerol shuttling into glycerol lipid synthesis rather than into de novo synthesis of glucose [74]. In cholangiocytes, AQPs were found to contribute to ductal bile secretion and formation, upon secretin stimulation for AQP1 [75], whereas AQP4 was expressed at the basolateral membrane, being secretin unresponsive [76]. Of note, AQPs are expressed in both apical and basolateral plasma membranes in cholangiocytes. In particular, AQP1 was shown to be responsible for the apical transport of water, whereas AQP4 was restricted to the basolateral membrane of cholangiocytes [77]. In AQP1-null mice, bile flow and bile salt concentration were unchanged, but dietary fat absorption was defective, whereas in rats, AQP1 seems to be pivotal in rat ductal bile formation [78,79]. In hepatic stellate cells (HSCs), the importance of AQPs in activation, apoptosis, and quiescence was shown by us and other groups. Interestingly, only quiescent HSCs express AQP0, AQP1, AQP5, AQP8, AQP9, and AQP12. The changes in AQP expressions were connected to an increased resistance to apoptosis in activated HSCs [80]. We demonstrated that AQP3 expression was driven by adiponectin stimulation in LX2 cells [81] through PPARα signaling [82], which had a profound effect on glycerol uptake, lipogenesis, and lipid storage, also downregulating HSC activation and fibrosis markers. We further explored the role of AQP3 in primary HSCs isolated from patients with non-alcoholic steatohepatitis (NASH), showing an upregulation of its protein and gene expression, which was proportional to the fibrosis stage, by PPARγ through the c-Jun N-terminal kinase (JNK) pathway [83].

## 5. Other Nuclear Receptors Involved in Systemic AQPs Regulation

FXR is also involved in AQP regulation in the kidney, playing an important role in renal water reabsorption [84]. Activation of FXR effectively lowered urine volume, and increased osmolarity, as evidenced by polyuria in FXR knockout animals. Furthermore, the FXR agonist, chenodeoxycholic acid (CDCA), upregulated renal AQP2 expression in wild-type (WT) animals, whereas a loss of AQP2 was found in FXR knockout animals. In vitro luciferase assays identified a FXR binding site in the gene promoter region of both human and mouse AQP2 [84]. Other studies highlighted the importance of LXR in AQP regulation. LXRβ KO mice showed augmented inflammation, pancreatic insufficiency, and reduced serum levels of amylase and lipase. WT mice expressed LXRβ in the nuclei of ductal epithelial cells, and AQP1 in the plasma membrane, which was undetectable in LXRβ KO mice. An LXR agonist also successfully increased AQP1 gene expression, highlighting a tight cross-talk between AQP1 and LXRβ. These data demonstrate that LXRβ, but not LXRα, controls AQP1 expression in the pancreas, thus linking dietary resistance to weight gain treatments, and pancreatic insufficiency due to impaired pancreatic exocrine secretion mediated by AQP1 [85]. Central diabetes insipidus was also connected to impaired expression of AQP1 in kidneys from LXRβ animals. LXRβ KO mice experienced polyuria and polydipsia (increased water intake), both key features of this disease. When the LXR agonist (GW3965) was used in WT mice, it triggered increased urine osmolality, suggesting LXRβ as a key NR controlling water balance, and thus, AQP1 being a possible target in water balance disorders [86]. In line with these results, other authors demonstrated that AQP2 expression was significantly reduced in renal collecting ducts of LXRβ knockout mice [87]. Upon LXR agonist treatment, AQP2 was induced in the inner medullary duct cells of primary cultured mice, suggesting LXR represents a pivotal regulator of body water homeostasis via AQP regulation [87]. The nuclear receptor regulation of AQPs is summarized in Table 2.

## 6. Clinical Relevance of AQPs

Only a few studies investigated the clinical relevance of AQPs in humans. In cancer, AQPs were shown to have fluctuating expression during the gap 1 (G1) phase, further demonstrating that AQP2 could decrease the transit time through the synthesis (S) and gap 2/mitosis (G2/M) phases of the cell cycle [88], also taking part in the control of the G1–S transition [89]. In hepatocellular carcinoma (HCC), Chen et al. discovered an upregulation of AQP3, while AQP7 and AQP9 were downregulated, correlating with tumor grade, stage, and lymphatic metastasis [90]. In fact, other studies proposed AQPs as possible therapeutic biomarkers and targets within cancer biology, with special interest in colorectal, liver, lung, brain, and breast cancers. However, whether they represent actual therapeutic and diagnostic molecules still remains questionable [91]. In another report, AQP9 expression was shown to be reduced in patients with non-alcoholic liver disease (NAFLD) and non-alcoholic steatohepatitis (NASH), translating its downregulation into a compensatory mechanism against TG accumulation in fatty liver [92]. In this context, gender differences should not be disregarded; for instance, AQP9 expression in hepatocytes was documented to be higher in males than in females [93]. In another study, after 90 min exercise, women had higher levels of plasma glycerol when compared with those of men, probably due to the reduced carbohydrate levels, and increased lipid utilization [94]. This difference may be explained by a higher expression of AQP7 in visceral fat in women, or by their increased adiposity when compared with that of men.

Furthermore, subjects with a genetic variant of AQP7 featuring a deletion of guanine at position 953 (−953G) had an increased risk of obesity and/or T2DM [95]. Others found a strong association between AQP7 and lipogenic/lipolytic genes in obese human AT, with a strong increase in AQP7 gene expression in visceral adipose tissue (VAT) from T2DM obese subjects [96]. Accordingly, another group demonstrated a strong elevation of AQP7 expression in VAT of obese patients, accompanied by a decrease in hepatic AQP9 in obese T2DM subjects [63]. It is, therefore, tempting to speculate that part of the beneficial effects of PPARγ agonists, glitazones, in NAFLD comes from the expansion of WAT trapping glycerol in TGs, increasing insulin sensitivity and deactivating liver AQP9 function [97,98], while the lack of effects of PPARα agonists, such as fibrates or elafibranor [99,100], was due to increased liver AQP9 activity, shuttling in glycerol and overloading fatty livers with lipid synthesis precursors, in the context of exacerbated de novo lipogenesis [101].

## 7. Conclusions

AQPs remain elusive pharmacological targets [20], perhaps due to their complex tissue-specific regulation. Mathematical models were recently developed to better decipher AQP physiology, and may represent useful tools in the future to understand the dynamics of glycerol, glucose, and energy homeostasis in metabolic disorders [102]. AQP coordination in the liver and AT is driven by PPARα and PPARγ, respectively, as proposed by a study from Patsouris et al. [103], although many AQPs seem to be responsive to both NRs upon tissue localization. Efforts should be made to develop selective blocking strategies in chronic disorders such as obesity, fatty liver, or skin diseases, in which biological evidences suggest a role for AQPs in pathogenesis.

## Figures and Tables

**Table 1:** Solute selectivity of aquaporins (AQPs), and their localization and disease implications.

AQP	Solute Selectivity	Localization	Disease Implications	Ref.
**AQP0**	Water	Eye	Cataract	[24]
**AQP1**	Water	Brain, kidney, red blood cells	Tumor genesis and metastasis	[25]
**AQP2**	Water	Kidney	Nephrogenic diabetes	[26]
**AQP3**	Water, glycerol, urea	Liver, kidney, skin, intestine, eye, red blood cells	Skin cancer	[27]
**AQP4**	Water	Brain, intestine, kidney eye, nose, salivary duct, placenta, muscle	Brain edema, epilepsy, neuromyelitis optica	[28,29,30,31]
**AQP5**	Water	Lungs, salivary glands, sweat glands, eye, pancreas	Asthma, cystic fibrosis	[32,33]
**AQP6**	Water	Kidney	Unknown	[34]
**AQP7**	Water, glycerol, urea	Adipose tissue, liver, kidney, testis, heart muscle	Obesity, T2DM	[35,36,37]
**AQP8**	Water	Intestine	Ulcerative colitis	[38]
**AQP9**	Water, glycerol, urea	Liver, red blood cells	Obesity, T2DM. NAFLD	[37,39,40]
**AQP10**	Water, glycerol, urea	Small intestine	Unknown	[41]
**AQP11**	Water	Liver, testis, kidney	Polycystic kidney disease	[42]
**AQP12**	Unknown	Pancreas	Pancreatitis	[43]

T2DM—type 2 diabetes mellitus; NAFLD—non-alcoholic fatty liver disease.

**Table 2:** Nuclear receptor (NR) regulation of AQP subtypes.

NR	AQP	Model	Ref.
**PPARγ**	AQP3, AQP7	Human HSCs; 3T3-L1 cells and mice treated with PPARγ agonist PGZ.	[47,83]
**PPARα**	AQP3, AQP7, AQP9	Human HSCs; PPARα null mice; rat treated with PPARα agonist—WY14643 and HepG2/WIF-B9 cells.	[48,74,82]
**FXR**	AQP2	FXR KO mice/treated with CDCA, and primary IMCDs cells.	[84]
**LXRβ**	AQP1, AQP2	LXRβ KO mice, treatment with LXRβ agonist in WT mice; LXRβ KO mice, IMCDs and mIMCD3 cell line.	[85,87]

PGZ—pioglitazone; IMCDs—inner medullary collecting ducts; FXR—farnesoid X receptor; LXR—liver X receptors; HSCs—hepatic stellate cells; CDCA—chenodeoxycholic acid; KO—knockout; WT—wild-type.
